# Extracting Safety-II Factors From an Incident Reporting System by Text Analysis

**DOI:** 10.7759/cureus.21528

**Published:** 2022-01-23

**Authors:** Takeru Abe, Hitoshi Sato, Kyota Nakamura

**Affiliations:** 1 Department of Quality and Safety in Healthcare, Yokohama City University Medical Center, Yokohama, JPN

**Keywords:** safety-ii, text mining, electric health records, reporting system, incident

## Abstract

Introduction

The use of electric health records (EHRs) has spread worldwide and has helped record huge amounts of data. However, despite accumulated data from EHRs, especially text data, the information has been underutilized. Our research questions and aims are as follows: How can an incident report system extract common themes behind incidents, good practices, improved quality, and safety based on the Safety-II/resilient healthcare approach?

Methods

We extracted data from the electronic incident reporting system of the Yokohama City University Medical Center between April 1, 2016 and March 31, 2018. We utilized natural language processing and text mining to extract concept categories and word patterns. We also used the incident levels as outcomes, as well as classification and regression tree analysis to obtain associated text combinations.

Results

A total of 17,231 cases were reported through the electronic incident reporting system in our hospital during the study period. Hospital staff has to be prepared for incidents with complex mechanisms in daily practice. The hospital staff tend to focus on individual actions rather than considering a systematic approach.

Conclusion

Certain combinations of professions and contents may contribute to resilient management. Studies on Safety-II management utilizing clinical information and text records are needed.

## Introduction

The use of electric health records (EHRs) has spread worldwide and has helped record huge amounts of data. However, despite accumulated data from EHRs, especially text data, the information has been underutilized [1]. An EHR can be used as a tool for incident reporting and as a patient clinical record system to improve quality and safety in healthcare.

The concept of Safety-II, originally developed for industrial quality and safety management, has recently been utilized for patient safety. It focuses on how things go right, along with the concept of Safety-I, which focuses on how things went wrong learning from the past [2-4]. Hypothetically, rather than only focusing on the 5% deviation to prevent accidents, the remaining 95% of the majority under the normal bell curve should be analyzed to promote good healthcare practices, as observed in the real world.

Among regular practices that work well, near-miss experiences can help establish a culture of safety and improve healthcare quality, as described in Good Catch Campaign [5]. In the Campaign, sharing information of near-miss experiences could lead to a reduction of incidents, accidents, or adverse events [5].

Thus, the use of EHRs to share near-miss experiences and, subsequently, to prevent adverse events could be a challenge and is yet to be proven. In this study, we report the preliminary results of utilizing EHR text data from an incident reporting system of a large educational hospital. Our research questions and aims are as follows: How can an incident reporting system extract common themes behind incidents, good practices, improved quality, and safety based on the Safety-II/resilient healthcare approach? We conducted a preliminary, exploratory, and mixed-method study utilizing the text data of an EHR.

## Materials and methods

Setting

In this study, we extracted data from the electronic incident reporting system of Yokohama City University Medical Center spanning two fiscal years between April 1, 2016 and March 31, 2018. The center has 726 beds and 18,000 inpatients annually. The population of Yokohama City was 3,740,944 in 2019, and there were nine critical care and emergency centers in the city. The population covered per center was approximately 415,660. The study was approved by the Institutional Review Board of the Yokohama City University Medical Center (B181000037). The Board also approved that the requirement for written informed consent was waived, and the individual informed consent was opted out, due to the nature of retrospective study design. The study was conducted in accordance with the principles of the Declaration of Helsinki, as well as per the Personal Information Protection Law and National Research Ethics Guideline in Japan.

Incident levels

The incident reporting system was anonymous and contained information regarding the incidents, such as the professions, location, incident level, free-text content regarding actual occurrence, and a measure to prevent future incidents. This information was entered electronically by a staff who witnessed the incidents. The seven nationally defined incident levels are shown in Table 1. A Japanese hospital must report to the Japan Council for Quality Health Care and publicly announce an incident that is Level 3b or above. In this study, we utilized data from Levels 0, 1, 2, and 3a for the analysis. We regarded Level 0, contrasting to 1, 2, and 3a, as a good catch based on Safety-II principle. In addition, we regarded Level 3a, contrasting to 0, 1, and 2, as an incident based on Safety-I principle.

**Table 1:** Reported incident levels in Japan

Incident levels	Descriptions
Level 0	Incident with no direct impact on patients
Level 1	Incident with no substantial damage to patients
Level 2	Incident with minor damage to patients requiring no further treatment
Level 3a	Substantial damage to patients requiring further treatment without prolonged hospital stay
Level 3b	Substantial damage to patients requiring further treatment with prolonged hospital stay
Level 4	Permanent disability related to accident
Level 5	Death related to accident

Text mining

Regarding the free text content, we used natural language processing and text mining [6-8] to extract concept-category and word patterns. First, we applied text information to dimensionality reduction and sentiment classification [9]. Second, we morphologically analyzed the co-occurrence among the classified words. We initially used co-occurrence network analysis to provide a graphic visualization of the potential relationships between words. Through the co-occurrence network, the presence of two or more words in the same text unit was extracted using the Jaccard distance [10]. Words that frequently co-occurred were connected by lines, based on the Fruchterman and Reingold layout algorithm [11]. Third, we tagged each category by the classification and added it as a binary outcome to use in the analysis described below. We utilized the IBM-SPSS Text Analytics for Survey 4 in Japanese (IBM Corp, Armonk, NY) and KH Coder® [12]. Lastly, we translated the output and results from the analysis into English for reporting.

Statistical analysis

**Figure 1:**
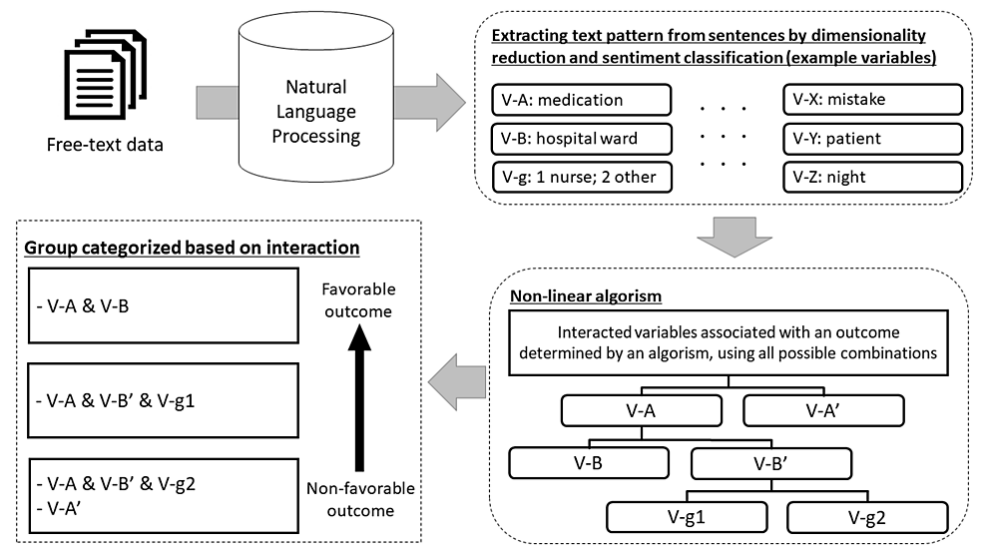
Novel scheme with text-mining and non-linear algorism Free-text data would be analyzed using natural language processing. Then, text or words patterns were extracted (the top, from left to right). Using the patterns, non-linear algorism could identify variable interactions, which are positively or negatively associated with an outcome variable. After applying the variable interactions, the total sample can be divided into mutually exclusive sub-groups with different combinations of text variables, and the groups can be ordered by a degree of favorable or non-favorable outcome (the bottom, from right to left). Image credits: Takeru Abe, Hitoshi Sato, Kyota Nakamura.

We used descriptive statistics for all the variables described above, including the categorized text information. Next, we used the incident level as an outcome and utilized a classification and regression tree (CART) analysis to obtain associated combinations of text. Identified variable combinations for Levels 0 and 1 were used for extracting factors of good practices. In addition, the CART is appropriate for exploratory analysis; especially since there are several candidate and unknown variables, higher-order interactions of various combinations are expected. Instead of continuous variables, CART analysis is utilized for categorical and independent variables [13]. The CART identifies subgroups that would be appropriate for our sample because this research aimed to identify combinations of variables, rather than to characterize a case with an odds ratio obtained from linear regression models. In addition, each case may have complex dynamic backgrounds and risk evaluation with a single factor may be difficult. Thus, an inpatient was categorized into a specific subgroup with certain combinations of factors, thus enabling us to extract information on the good practices and precautions. The analysis procedure and scheme of this study is described in Figure 1.

Hierarchical cluster analysis was then used to extract common themes of reported measures against incidents from the written content. The clusters were obtained as groups of words that provided common themes for each cluster.

We used the IBM-SPSS Statistics 25.0. (IBM Corp., Armonk, NY) for all statistical analyses. Statistical significance was set at p < 0.05.

## Results

A total of 17,231 cases were reported through the electronic incident reporting system in our hospital during the study period. On average, each staff member reported five to six cases annually. Table 2 shows the frequency in each incident level categorized by profession. Nurses comprised 89% of all reports (15,261/17,231). Levels 0 and 1 accounted for 88%.

**Table 2:** Incident levels and type of professions

Type of professions	Level 0		Level 1		Level 2		Level 3a		Total	
	frequency/%		frequency/%		frequency/%		frequency/%			
Nurse	2,742	18%	10,684	70%	1,530	10%	305	2%	15,261	89%
Radiation technician	232	40%	319	55%	21	4%	6	1%	578	3%
Physician	186	35%	218	41%	74	14%	49	9%	527	3%
Pharmacist	235	82%	48	17%	3	1%	0	0%	286	2%
Laboratory technician	56	37%	92	61%	2	1%	0	0%	150	1%
Resident	69	58%	35	29%	11	9%	5	4%	120	1%
Other	98	32%	177	57%	24	8%	10	3%	309	2%
Total	3,618	21%	11,573	67%	1,665	10%	375	2%	17,231	-

**Figure 2:**
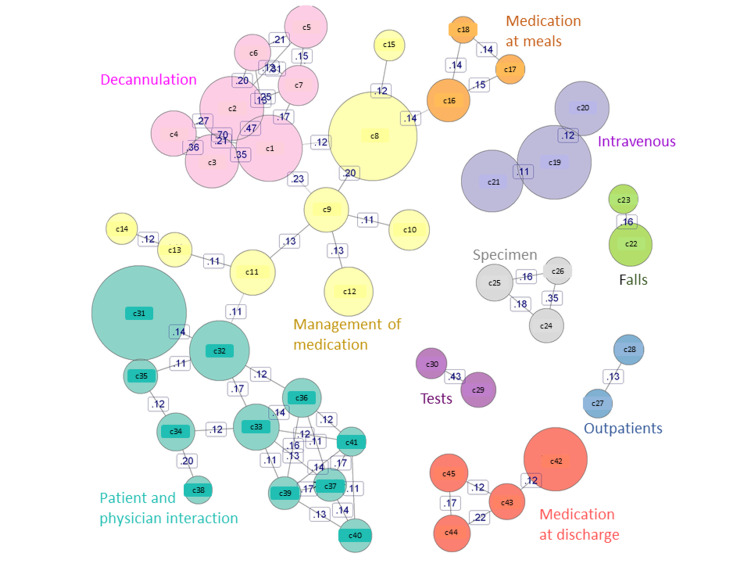
Text-mining and identified co-occurrence from incident reporting Correlation of reported words obtained from co-occurrence network analysis. The identified groups were as follows: patient and physician interaction, decannulation, management of medication, intravenous administration, specimen, falls, medication at meals, medication at discharge, outpatients, tests; c (Co-occurrence) 1. own-self, c2. pull, c3. extubate, c4. stomach, c5. line, c6. venous, c7. needle, c8. internal use, c9. management, c10. internal agent, c11. nursing, c12. error, c13. ward, c14. communication, c15. overdose, c16. medication, c17. supper, c18. breakfast, c19. administered, c20. intravenous drip, c21. order, c22. falls, c23. toilet, c24. specimen, c25. urine, c26. submit, c27. examination, c28. outpatients, c29. measurement, c30. glycemia, c31. patient, c32. check, c33. physician, c34. report, c35. self, c36. implement, c37. ordering, c38. receive, c39. imaging, c40. mistake, c41. change, c42. forget, c43. provide, c44. discharge, c45. prescription. Image credits: Takeru Abe, Hitoshi Sato, Kyota Nakamura.

From text-mining and identified co-occurrence, the word “patient” and “after” were counted 150 times, the most frequent connection among other co-occurrences. The word “medication” and “after-the-meal” connected 100 times. Along with the word “patient,” it had 56 connected words, and “checking” was the most frequent (400 times). Figure 2 shows the correlation of the reported written words obtained from the co-occurrence network analysis. Regarding incident contents, the identified groups were varied as follows: (a) patient and physician interaction, (b) decannulation, (c) management of medication, (d) intravenous, (e) specimen, (f) falls, (g) tests, (h) medication at meals, (i) medication at discharge, and (j) outpatients.

**Figure 3:**
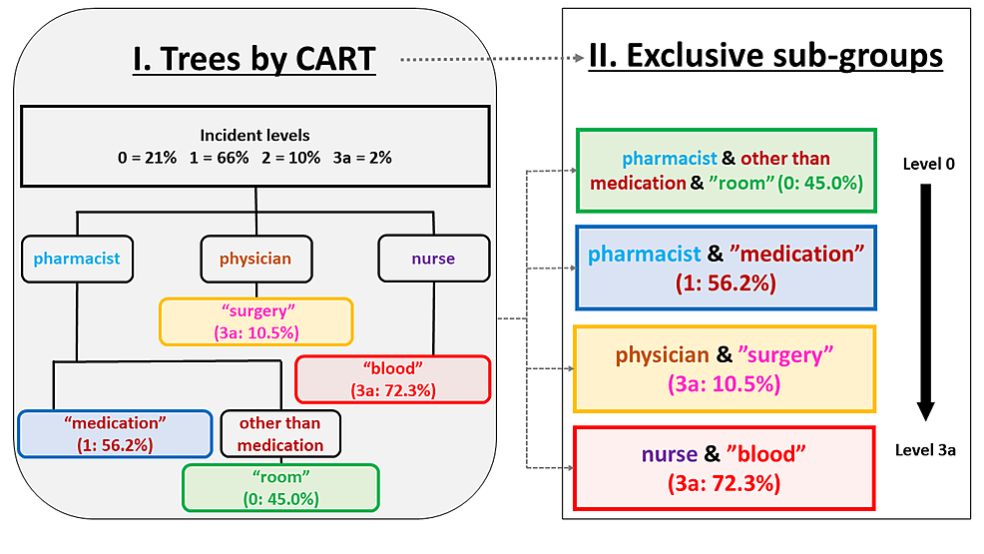
Multiple-layered tree associated with the incident level Multiple-layered tree associated with the level of incidence. The most significant variable was the type of profession. CART analysis identified four exclusive subgroups of cases based on the significant proportion in each incident level. Image credits: Takeru Abe, Hitoshi Sato, Kyota Nakamura.

Figure 3 describes a multiple-layered tree associated with the incident level. The most and the first significant variable was the profession. Each profession had different word variables, such as “medication,” “blood,” or “room.” Furthermore, a combination of a pharmacist, the word category without “medication,” and the word “room” had the highest proportion (45.5%) for Level 0. A combination of a pharmacist and the word “medication” had the highest proportion (56.2%) for Level 1. Among physicians, the combination of the word “surgery” had the highest proportion (10.5%) for Level 3a. A combination of a nurse and the word “blood” had the highest proportion (72.3%) for Level 3a. CART analysis identified four exclusive subgroups of cases based on a significant proportion of the incident levels.

**Figure 4:**
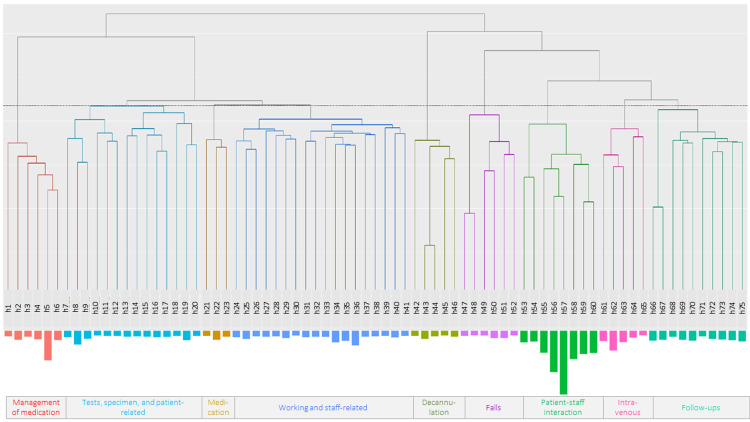
Common themes in the reported measures against the incidents The identified groups were the following: patient and physician interaction; work- and staff-related; tests, specimen, and patient-related; follow-ups; management of medication; decannulation; falls; medication; intravenous; h (Hierarchical cluster) h1. tablet, h2. own, h3. internal agent, h4. medication, h5. internal use, h6. management, h7. repeatedly, h8. self, h9. explanation, h10. cancel, h11. planned, h12. current day, h13. admission, h14. discharge, h15. request, h16. ward, h17. communication, h18. urine, h19. testing, h20. blood withdrawal, h21. box, h22. agent, h23. prescription, h24. work, h25. in-charge, h26. day shift, h27. change, h28. written, h29. checking, h30. reveal, h31. notice, h32. forget, h33. later, h34. implement, h35. before, h36. doing, h37. hereafter, h38. receive, h39. surgery, h40. possible, h41. enter, h42. fix, h43. pull, h44. extubate, h45. control, h46. stomach, h47. call, h48. nurse, h49. by, h50. measurement, h51. bed, h52. falls, h53. measures, h54. consider, h55. nursing, h56. patient, h57. confirmation, h58. order, h59. physician, h60. report, h61. intravenous line, h62. administered, h63. intravenous drip, h64. begin, h65. finish, h66. process, h67. observation, h68. night shift, h69. response, h70. time/hour, h71. condition, h72. finding, h73. visiting, h74. use, h75. necessity. Image credits: Takeru Abe, Hitoshi Sato, Kyota Nakamura.

Figure 4 describes the common themes in the reported measures against the incidents. Identified groups were varied as follows: (a) patient and physician interaction, (b) decannulation, (c) management of medication, (d) intravenous, (e) specimen, (f) falls, (g) tests, (k) medication, (l) working and staff-related, (m) patient-related, and (n) follow-ups.

## Discussion

The study identified combinations of professions and contents of work activities as contributing factors for good practices, as well as four exclusive subgroups associated with incident level. Nurses, pharmacists, and other medical staff may have contributed to the safe environment by constantly reporting good practices. In addition, various themes of incidents were extracted, which indicates that the hospital staff must be prepared for incidents with complex mechanisms in daily practices. Regarding measures to incidents, the hospital staff focuses on individual actions rather than a systematic approach. This may be affected by Japanese cultural norms. However, there is an opportunity to educate the staff to apply a systematic approach for each case, that is, systems approach or human factors. This can reduce the double-checking behavior, which would subsequently lower the workload and incidents. The findings can only be applied to a larger set of data and a more validated methodology, such as analyzing texts with clinical information from medical records.

Our findings suggest that pharmacists attempted to create a culture of safety because their reports contained higher proportions of Levels 0 and 1. Promoting a near-miss or good-practice report contributes to a safe environment and fewer adverse events [5]. Nurses and physicians used the reporting system effectively because they reported Level 3a incidents more frequently out of clinical necessity and responsibility. A culture of safety may be promoted if templates or samples of near-miss and good practice are disseminated. In addition, the study applied an approach with text mining and non-linear statistical analysis, which was not frequently done in healthcare area. Our findings could provide a novel scheme to analyze a large dataset including free-text data, which could be subjective but reflect a real world.

Our approach to free-text content is novel in the field of quality and safety research. While applying text mining has been well established [6-8], analyzing co-occurrence relies on subjectivity. On the other hand, our use of CART analysis addressed the limited interpretation and produced findings that are highly reliable and generalizable. Additionally, CART analysis can identify non-linear relationships from several variables [9]. Our use of text data on CART analysis revealed the associations of various unknown factors.

This study has several limitations. First, this was a single-center study, and the external validity or generalizability of the findings was limited. Thus, a multi-center study should be needed to validate the study findings. Second, since reporting may be affected by individual writing skills, the reliability of the findings may also be limited. Standardization of reporting or establishing a set of questions may be needed to address the lack of internal validity. Third, since we did not include the patients’ clinical information as variables, the validity of the findings could also be limited. Further studies are needed to address these limitations. Fourth, we utilized text mining in Japanese and then translated the output into English. Translation of text information into English before analysis may lead to inconsistent word connections. It is also difficult to consider cultural values during translation. Thus, translating outputs might be less prone to ignoring cultural differences. Fifth, we did not evaluate the contents of incidents with other common methods, such as systems approach [14,15] or human factors approach [16]. A future study with mixing approaches might be needed. Lastly, this was an exploratory study that attempted to extract an element of Safety-II from the text information of an incident reporting system of a university hospital. Findings should be presented to the staff and evaluated in clinical settings in a future study.

## Conclusions

In this study, we conducted a preliminary, exploratory, and mixed-method study utilizing the text data of an EHR. We identified a multi-layered structure of reports and characteristics in relation to incident Level 0. Nurses, pharmacists, and other medical staff may have contributed to the safe environment by constantly reporting good practices. Various themes of incidents were extracted, which indicates that the hospital staff must be prepared for incidents with complex mechanisms in daily practices. Further studies on Safety-II management utilizing clinical information and text records, as well as a multi-centered study, are needed to validate findings.
